# Protrusion of the Upper Jaw

**Published:** 1894-10

**Authors:** R. Ottolengui

**Affiliations:** New York City.


ARTICLE IV.

### PROTRUSION OF THE UPPER JAW.

BY R. OTTOLENGUI, M. D. S., NEW YORK CITY.

Time was, and not so very long ago, when an expert in the regulation of teeth might expect to have nearly all classes of irregularities referred to him by his brother dentists. With the advance of time and an increased knowledge of the technics of the subject, more men have essayed to regulate the teeth of their patients. At first they treated simpler cases, where the tortion of a single tooth, or the reduction of two or more to their proper places within the arch, offered few obstacles to success. But, gaining courage with experience, the general practitioner has widened the sphere of his endeavors, until the specialist nowadays receives from him only those very difficult cases, which with all his increased knowledge he dreads to undertake.

It is interesting to inquire into the nature of these deformities, so extreme in character that really expert men send them elsewhere, and yet which can be corrected, since the specialist accepts and treats them. I should divide them into two classes, the first of which would include unique and excessive departures from the normal mould, and the second, merely protrusion of the upper or lower jaw.

Thus it may be held as true, not considering the most horrible freaks of nature, and dealing exclusively with such deformities as may be classified into groups because of their frequent occurrence, protrusion of one of the jaws is the condition which at present marks the limitation of the skill of the general practitioner.

When the specialist receives a letter from a dentist, perhaps a thousand miles distant, stating that he has sent a patient, it would be reasonable to wager ten to one that the case, when presented, will prove to be a protrusion, and ninety per cent. of all such, fortunately, will be of the upper jaw. For these reasons have I chosen this class as one that may well be dealt with apart from the general subject of irregularities.

Before beginning any discussion of the methods of treatment, let me say a word about etiology. There are some who find it profitable to spend much time and labor upon speculations as to the why or the wherefore of this, that, or the other aberration. They try to find causes for every twist or prominence that departs from the normal type. To me this seems labor lost, or almost lost. Of course it would be interesting to know positively why Master Jackie's upper jaw protrudes, when his brother Johnnie has a perfect arch and occlusion, and his sister Sue, *perçontra*, protrudes the lower jaw, notwithstanding the fact that all three have the same mother, and presumably the same father. I say it would be interesting, but out of all the mass of theorizing that has helped to pad out our journals and text books, what single fact in this connection has yet been proven—proven beyond dispute, I mean; proven so that it has been accepted as a final doctrine, forever?

And then, to view this from another aspect, what difference does it all make to Jackie? He wants his protruding upper jaw retreated—that is what he wants. His sister Sue wants the same thing done to her lower jaw. Little Johnnie, lucky boy, snaps his fingers at the whole crew of etiologists, and says: "I'm the same as Jackie and Sis, so far as my etiology goes, but my teeth are all right, thank you!" But these two children, with their crooked jaws and distorted features, are in our hands for assistance. Suppose the etiologist really solves the problem of cause, mixed and muddled as it is; does that aid the dentist one jot in his work? Is his treatment and management of the case to be affected in any way by what the etioligist tells him?

I think not. I would take Jackie's case without inquiry into his previous history; without caring whether he sucked his thumbs or his mother's breast too long; without knowing, or wishing to know, whether he had a great aunt, five degrees removed, who sucked her thumbs and protruded her jaw. All such considerations are worthless when one comes to the practical task of correcting the deformity.

The one argument that might be made is that if the case is what has been termed congenital, it will be more resistant; that is, that there will be an unusual effort made toward a return to the irregularity, indicating a longer necessity for the retaining fixture.

But even in this connection we do not need the etiologist to teach us what we must do, and I even doubt whether the character of the cause of the irregularity has anything whatever to do with the undoubted fact that the retaining fixture must be persistently worn. This will be true whether the case be congenital or not. I may consider this point as well here as elsewhere.

Suppose for a moment that a girl presents, with the four superior incisor teeth biting *within* the lower arch. Suppose, if you like, that her sister, her brother, her father, her mother and her mother's mother all have, or have had, the same abnormality. That would be a perfect case for your etiologist, and the claim of congenial aberration would perhaps be well founded. Then it should follow here as elsewhere that if regulated there would be a great effort to return to the irregular position. But suppose that these four incisore be regulated so that they bite properly, outside the lower arch, will they return? Certainly not. Provided that they do bite well over the outside of the lower arch, no retaining fixtures will be required at all, after a reasonable period has been allowed for the fixation of the teeth in their sockets. Why? Because the lock of the new. but normal occlusion, tends to hold them out. Also because the act of incising food tends to protrude the upper teeth, if it affects them at all.

With a protruding arch, which has been retracted, it is quite otherwise. The teeth forcibly carried backward, finally impinge upon the lower teeth, as in the other case, but now the latter can have no influence restraining them from returning to their former places. It may be that the alveolus has been crushed together, rather than absorbed, and, acting like a compressed sponge, tries to expand again, and in this way carrying the teeth forward once more. And here, certainly, and what is most important, the new bone formed may well be inadequate to hold the teeth. In the movement *Outward*, new bone formed *behind* the teeth is solidly re-enforced by the entire jaw back of it, thus acting as a permanent restraint to the return. But where the teeth are *retreated*, the solid part of the bone is either compressed or absorbed to allow the movement, and the labial part of the alveolus goes in with the teeth. This labial bone cannot act as a restraining cage to the teeth, until it has been firmly knitted to the palatal part of of the socket by a solidification of that which fills the spaces between the roots. Now the whole alveolar bony tissue is exceedingly spongy and soft until quite beyond the age at which we usually regulate teeth. Therefore, regardless of congenial circumstances, it will usually be necessary to hold the teeth in place for an indefinite period. Again, the incising of food during mastication, if anything must work against their retention rather than otherwise.

How then shall we decide when it may be safe to remove the retainer? It is usually my custom to instruct the patient to wear the restraining cage or band for one year, night and day. In some cases I make a cage which is cemented on for a period of three months, that the solidification of the bone around the teeth may be more thoroughly accomplished. Then substitute a removable fixture. At the end of a year or earlier, at the discretion of the operator, the restrainer may be worn at night only, and during meals, the orders being that if at any time it should be found difficult to replace, (a signification that a forward movement had been iniatiated) then the restrainers should be again worn continuously. Even after nightly wear during another year, it would be wiser to abandon it on alternate nights than to throw it aside entirely. I have known a case where an instrument was worn in this way for five years, and two years later there is a slight protrusion which shows a space at the median line. The cage was abandoned too early in this case, which, by the way was not congenital so far as the history shows. To conclude this part of my argument I have only to say that however valuable the etiology of irregularities may be as a contribution to science, so far, at least, nothing has been proven which in any way known to me facilitates the correction of oral deformities connected with the teeth.

DIAGNOSIS.

In undertaking the care of a prominent superior jaw, the diagnosis is of primary importance. The jaw may be actually prominent,or apparently so. The fact that the superior incisors overlap the inferior by a quarter of an inch or more, by no means proves that the upper jaw is abnormal. The fault may be in the lower jaw entirely. To decide this point, the countenance should be considered by examining the profile, with the lips at rest. At once it may be noticed that in this pose the lips do not meet, the teeth showing between. If the upper lip be short, arched upward, and the upper teeth taking a similar direction, correction may be confined to the upper jaw. If the lower lip curl inward, hiding the lower teeth, which also incline toward the tongue the abnormality may be exclusively in the lower incisive region. Before opening the mouth to examine the teeth, note whether the distortion of the features depends upon a prominent superior maxilla, which in extreme cases may even make the nose itself appear turned up. Also observe whether the whole chin seems to be retreating, so that the facial contour would be perfected by moving it forward. If the chin seems to recede, have the patient slide it slowly forward without opening the mouth, and at various points decide where it should rest in order to effect the most æsthetic results.

During this examination, it may be found that—

(*a*) The frontal alveolus of the superior maxilla is too prominent; the lower jaw is a normal pose. Treatment indicated is a reduction of the frontal prominence of the upper jaw without interference with the lower.

(*b*) Superior frontal prominence and inferior frontal recession, confined in both instances to the alveolar process. Treatment: Reduction of upper prominence, and forward movement of lower incisive region.

(*c*) Superior maxilla normal; inferior incisive region retreating. Treatment: Correction of the deformity of lower incisive region.

(*d*) Frontal prominence in the upper jaw, combined with a narrowing in the bicuspid region; lower jaw normal. (This, however, would be unusual, as a narrowing of the superior jaw similarly, with a crowding in the incisive region. This, because however irregular the teeth, we commonly find an attempt at occlusion.) The treatment indicated is to widen the jaw (or jaws) and reduce the prominence, the first making the second possible.

(*e*) A frontal prominence of the upper jaw; lower jaw normal, so far as the shape of the dental arch and the pose of the teeth are concerned, but the entire set of teeth occluding one-half tooth or more posterior to a typical occlusion. Treatment necessitates a reduction of the prominence and a correction of the false occlusion, with restoration to proper facial contour, by "jumping the bite."

(*f*) Superior end inferior arches both normally regular, but an apparent superior prominence, which is really an inferior recession, caused by a false occlusion of the jaws, the lower biting back of the typical occlusion. Treatment: Entire restoration of contour and correction of false occlusion by "jumping the bite."

### MODES OF TREATMENT.

In briefly outlining the management of the above described cases, I desire to state that after giving due attention and study, as well as trial, to the various methods suggested by others, involving spring pressure, tension by screws, etc., I am convinced that the best force is that obtained from the elastic band skillfully and wisely applied. There is no class of cases which more urgently demands that the work should be accomplished as rapidly as is consistent with safety. I need to look no further for an argument against the methods which I deprecate, than a examination of the dates on the models which their advocates exhibit at dental meetings and clinics. By counting the elapsed time from the beginning to the conclusion of these cases, I almost invariably find that from two to even ten months more than necessary have been consumed. A case which I saw at a meeting once, exhibited with much pride by the operator, had occupied from January one year to February of the next, and so it happened in an exactly similar case, the patients being of the same sex and age, had also been dismissed by us in February, which had only been begun in the latter part of the previous November. It is, therefore, for this reason that I adhere to the old-fashioned rubber band, in preference to the new- fangled notions.

I have no patience with the men who are constantly striving to present new methods, or, as they are prone to term them "systems," of regulating teeth. These men are more anxious to advocate a "system," which they chisten with their own names, than they are to produce the best results for their patients. If there is a plan comprehensive and simple, which accomplishes a purpose satisfactorily in three months, there is no value whatever to a new system, however ingenious theoretical, and however beautiful on paper, if it requires double the time in practice. Nowhere in the whole realm of dentistry are there more false claims set up than in that department which regulates teeth. It is simply astonishing how many cases which have been regulated by the new systems require re regulating by the old. I recall especially one young lady who came to us for examination. She wished her teeth filled, but we did not understand at first the purpose of her visit, and she was advised that her teeth could be regulated, despite the fact that she had come so late in life, being then eighteen. In amazement, she said: "But my teeth have been regulated. Dr. Blank spent a whole year, and my father spent a thousand dollars to have it done. He says they cannot be improved." Yet, in spite of the learned gentlemen's statement, the upper teeth in the incisive region extended beyond the lower three sixteenths of an inch. Three months later that upper jaw was in perfect occlusion with the lower.

In the first case cited under the caption "diagnosis," the description is "the frontal alveolus of the superior maxilla is too prominent; the lower jaw is a normal pose. The treatment indicated is a reduction of the frontal prominence of the upper jaw without interference with the lower." By the last statement is meant, that the lower jaw being in proper pose, both as regards occlusion and outward facial contour, it must not be disturbed, but attention must be exclusively given to the upper jaw.

Having made the general diagnosis which classifies the case the next step is to make a more minute examination, in order to decide upon the details required in pursuit of the object. It is easy enough to say that treatment involves a reduction of the prominence, but how is this to be done? The chief point to be decided at this point is whether extraction be necessary or not. All of the permanent teeth—of course excepting the wisdom teeth—being in place, the child perhaps fourteen years old, I would think extraction advisable, provided the arch is generally symmetrical and all the teeth in close contact. I wish to be distinctly understood throughout this paper that in each instance the advice given is exclusively applicable to the special conditions indicated. In this case, we have started with the premise that the lower jaw is not to be disturbed, not requiring it. Under these circumstances

I say again, if the upper jaw be generally symmetrical and the teeth in contact throughout, the best and quickest, if not the only way to accomplish our purpose, is to extract two teeth. All things being equal, the choice should fall upon the first bicuspids, the reason for which is this: First, by so doing we have two teeth less to move than were we to extract the second bicuspids; and secondly, by leaving in the first bicuspids we not only add to the resistance of the body to be moved, but we take away from the anchorage of our force. To make this plainer, suppose that the twelfth-year molars are sufficiently advanced to permit an anchorage in the shape of a clasp or band thrown around them. With the first bicuspids out, we have six single rooted teeth to be moved, and four molars and two bicuspids to serve as resistance to our force. With the second bicuspids out, we have eight teeth to move and only four teeth resisting force. It might be argued that the teeth could be carried back two at a time, but this method is not only long and tedious, compared to moving all at one time, but it requires constant renewals of fixtures, and in the end, before completion, the long-continued strain will have loosened the anchor teeth, so that even the force required to move the heretofore untouched central incisors, the last in the series, will be more than they can withstand. Then, either we must abandon the work until the anchor teeth become firm, or else some extremely dificult method must be adopted, such as using a head-piece, or banding all the teeth that have been moved, and by thus uniting them obtain (perhaps) sufficient resistance to move the incisors.

Of course, if the patient presents with the first bicuspids sound and healthy, while the second have pulps exposed or otherwise worthless, that might be sufficient excuse for extracting the second bicuspids. Ordinarily, however, the first bicuspids are the best for the sacrifice. Before leaving this subject of extraction, let me say that if the case presents before the eruption of the canines, even though the correction is not begun at once but must be postponed for some months, it will be eminently wise to extract the first bicuspids. In a case of this nature that passed through my hands I advised such a procedure, and when the case came to be treated a year later the cuspids, had erupted quite near to the second bicuspids, and the course of treatment was greatly facilitated and abbreviated on that account.

Immediately upon the extraction of the teeth, except in the instance cited where the cuspids have not erupted and it is inconvenient for the patient to have treatment begun, the fixture should be inserted. Commonly all that will be required is a vulcanite plate, accurately adapted to the roof of the mouth, with clasps around the posterior sides of the second molars, and hooks in the plate opposite the palatal sulci of the same teeth. Another hook appears on the outer end of each clasp. Then a T-band is fitted about the labial surfaces of the six front teeth, and a hook is at each end. The stem of the T passes between the centrals, and also terminates in a hook. From this latter a rubber band is extended to each of the hooks on the plate and along the buccal side of the teeth an elastic band connects the hook at the end of the arm of the T with the hook which is on the end of the clasp. With exactly such a fixture I have seen many cases completed within three months, the six front teeth being carried back until the cuspids touched the second bicuspids. Elongation of the incisors during the progress is prevented by little hooks passing over the incisive ends, which also keep the T from pressing up against the gums.

Where the patient is younger and the anchorage to posterior teeth is not strong enough, then the prominence can be reduced by using a leather skull-cap, in connection with a bit which envelopes the teeth to be moved, arms extending out of the mouth for attaching the rubbers to the cap. In this way the back of the head becomes the point of resistance.

But it is not always necessary to extract teeth. The case may present where the teeth are not in contact, the four incisors especially flaring outward and away from each other, so that spaces occur all around. In such a case, the fixture described above will accomplish everything without extracting, because the teeth, when retreated so that they are in contact, will be found to be in good position. In such a case, if after reduction it is found that the teeth are still slightly in advance of the lower teeeth, but that the general effect is good, the deformity having disappeared, then it would be rash and unwise to extract two sound teeth merely for the gratification of carrying the upper teeth back a little more, for in such a case it would be manifestly impossible to reduce the arch until the cuspids touch the bicuspids, and unless this can be done so that the integrity of the arch may be restored, extraction is never advisable. If spaces be left in the arch, disaster is almost sure to follow later in life, by the tipping of the teeth adjacent to the space.

The next condition cited was a combination of a protruding upper jaw and a recession of the lower in the incisive region. Under these circumstances it would so seldom be necessary to remove any teeth that the regulation should be attempted without, and carried to a point where either success was attained or else it became manifest that a perfect result would be had only by extracting. This, because a part of the apparent overhang would be obviated by the forward movement of the lower incisors. These teeth could be brought out to a band, with rubber ligatures, but here if anywhere the spring wire becomes serviceable. It is more difficult properly to manage a band around the lower teeth than around the upper. The band, to afford sufficient resistance, must be sufficiently far from the labial surface of the teeth to cause a stretching of the rubber, and this will not be as readily tolerated by the lower as by the upper lip. Again, it is more difficut to use the rubber ligature on the lower incisors, because they are usually bathed in saliva. With syring wire, whether of steel or gold, the wires are tied to the teeth, their spring being at tension, and thus lying against the teeth they do not fret the lip, while the saliva acts helpfully on the linen ligatures by shrinkage.

In the third condition, the superior jaw being normal incisors retreating, the correction has to do only with a forward movement of the lower incisors, which would be the same as in the last case.

The fourth presents a frontal prominence in the upper jaw, with lateral narrowing across the bicuspid region. The treatment here is theoretically obvious. Suppose that the arch were a piece of wire bent exactly to conform to the curve of the alveolus; then imagine the ends of the wire fixed; it is evident that if the contracted sides are carried out, the prominent central portion must be correspondingly reduced. In the mouth this movement will not be coincident, because the arch is not a continuous material, but made up of separate teeth, wh ch must be operated upon individually. Nevertheless, the principal is the same. The narrowed sides must be carried outward, thus making it possible to reduce the frontal prominence.

It is very much easier to advise the widening of an arch than it may be to accomplish it. I have heard that it can be accomplished with piano wire bent and attached to a split plate, but that plan has never accomplished enough in my hands to recommend it. Any movement was so slow, that in some cases I was unable to afford the time, while in others the very slow action was certain to overcome and prevent my object, as I will explain.

Where the widening required is to be no more than half the width of a bicuspid, which is considerable, a jack-screw and split plate will usually accomplish all that is needed. But even so simple an instrument as this will, not infrequently, demand all the ingenuity of the operator to accomplish the purpose. The most common difficulty will be that the teeth are short, and their palatal surfaces so oblique that as soon as the screw is tightened so as to impinge, the plate is forced out of place, and therefore accomplishes nothing. On the other hand, it sometimes occurs that the bicuspids actually tip inward, offering oblique sides in such a direction that tightening the screw drives the plate up against the gums, so that the membrane will slough before the pressure moves the jaw bone, for it is the alveolar process, and not merely the teeth, that we wish to move.

When the slant of the palatal surfaces is such that the plate is forced out, this is to be overcome in various ways, according to circumstances. Sometimes properly constructed clasps will serve the purpose; or it may be necessary to tie the plate in, a method to be avoided if possible, because of the damage that may be done to the gums, causing recession, if nothing worse. Sometimes spurs of gold project between the teeth far enough to be caught under the "knuckle," will offer sufficient resistance. Another plan is to cement a gold band on a tooth on each side of the mouth, and place projections on the band at the palatal side, so that the plate rests under them and is so held. Indeed, there are these and many other ways of preventing the plate from leaving its place, but as the choice of either would require an amount of knowledge which would enable the operator to overcome the difficulty himself, I need not describe any other. The truth is, one cannot hope to give dogmatic methods for regulating which will be successful in the hands of all. The man who lacks the ingenuity to construct possibly new contrivances to meet special difficulties, need not hope to succeed in this department.

Where the slant is such that the plate is driven up against the gums too hard, it is to be obviated by a T, extending from each side of the plate, passing between the bicuspids, the arms of the T resting in the sulci of the bicuspids. This offers a resistance to the upward pressure of the plate, and prevents wounding the gums.

But sometimes it will be found that so slight a widening of the arch will not suffice for the reduction of the frontal prominence, and that the alveola cannot be pressed further out because of the fact that the bicuspids, and perhaps the molars, will slant so badly that they would offer their palatal services for occlusion with the lower teeth. If this result can be anticipated, then the plate for widening the jaw may be properly constructed from the start, and so more readily meet the requirements.

And now I come to the case in which the slow progress made with piano wire, or springs, defeats the end in view. In order to obtain the needed width, and at the same time retain the bicuspids and molars in their erect position, so that they offer their crowns for occlusion, it becomes advisable to separate the superior maxillæ along the central suture. This is accomplished by a plate, either cut entirely in halves, or else so nearly divided that there is only a slight connection at the posterior margin. The jack-screw is as short as possible, and set as high in the roof as it can be, and the plate itself, or extension thereof, comes forward so as to impinge upon the incisors, a clarp encircling the laterals or the centrals, as may be best. Then the screw is tightened twice a day, until the bones separate In young patients I have seen this occur within seven days, and two weeks ought to be the extreme of time required. In this way it is evident that as both jaw bones are moved apart, the teeth and their sockets are not disturbed at all, and the pose of the teeth remains the same.

It becomes immediately necessary to a very accurately fitting plate, to cover the roof of the mouth, and stiff enough to prevent the return of the jaws to their original position. The opening along the suture rapidly fills with bone, and there is absolutely no ill effects to be anticipated. The reduction of the arch may then be attempted by use of the long armed T already described.

In the last two cases the only procedure advised which has not been already considered, is that which has been so much discussed recently, viz.: "jumping the bite." A few men, most prominent among the number being Dr. Talbott, of Chicago, have tried to prove that the operation is impossible. They have descanted learnedly about the relation of the jaw to the articulation, in the compulsory new pose. All sorts of irrelevant theories have been advanced to show that the jaw cannot and will not, adopt a new closure. All this talk undoubtedly will create a doubt in the minds of those who know nothing about the matter, and who, having never undertaken the operation, are as ready to believe one side as the other. It also serves a a satisfactory purpose to those who, having made the attempt, have failed, as Dr. Talbot tells us he has, using that failure on his part as one of the arguments against the possibility. This argument really amounts to no more than this, in concise language: If one man finds that he cannot do it, it must be impossible.

But of what avail is all this theorizing with those who have tried it and have succeeded? When I say it is possible to "jump the bite," I know that it can be done, because I have done it, not once, but many times. And my success is a positive argument in favor of the method, which is much stronger than Dr. Talbot's failure, which serves as a negative sort of proof. If one man performs a feat he answers a thousand failures.

As to the method of accomplishing this, it is as follows: The frontal prominence of the superior jaw having been reduced as much as possible, the lower jaw is moved forward to a good occlusion with the anterior part of the jaws, little consideration being given to the posterior teeth. The lower jaw must not be progressed, however, beyond what becomes a part of the best facial contour, especial observation being given to the pose of the lips, and the relation of the chin to the rest of the features. As soon as it is decided just where it is most desirable to have the lower jaw, a plate is made which snugly fits the roof of the mouth, and which has, at the anterior part, an inclined plane, which not only prevents the closure of the mouth in the old position, but by catching the tips of the lower teeth, causes them gradually to slide forward in closing, so that the mouth shuts in the desired pose. This plate is worn until the habit becomes fixed, or until the retaining fixture is finally abandoned.

The bite may become a new habit in two or three months, and I have seen a child adopt it in less time, without the inclined plane, and with nothing whatever to produce the change except an indomitable will power, and sufficient interest in her own welfare for her to second the efforts made in her behalf.—*Dental Practitioner and advertiser.*

